# Novel linezolid loaded bio-composite films as dressings for effective wound healing: experimental design, development, optimization, and antimicrobial activity

**DOI:** 10.1080/10717544.2022.2127974

**Published:** 2022-10-02

**Authors:** Dina Saeed Ghataty, Reham Ibrahim Amer, Reham Wasfi, Rehab Nabil Shamma

**Affiliations:** a Department of Pharmaceutics, Faculty of Pharmacy, October University for Modern Sciences and Arts (MSA), Giza, Egypt; b Department of Pharmaceutics and Pharmaceutical Technology, Faculty of Pharmacy, Al-Azhar University, Cairo, Egypt; c Department of Microbiology and Immunology, October University for Modern Sciences and Arts (MSA), Giza, Egypt; d Department of Pharmaceutics and Industrial Pharmacy, Faculty of Pharmacy, Cairo University, Cairo, Egypt

**Keywords:** Linezolid, I-optimal design, bio-composite films, antimicrobial activity, wound healing

## Abstract

Biphasic release bio-composite films of the low water-soluble drug, linezolid (LNZ), were formulated using the solvent casting technique. Different polymers and plasticizers (gelatin, Tween 80, polyethylene glycol 400, and glycerol) were assessed for the preparation of bio-composite films. An I-optimal design was applied for the optimization and to study the impact of polymer concentration (X_1_), plasticizer concentration (X_2_), polymer type (X_3_), and plasticizer type (X_4_) on different LNZ-loaded bio-composite films. The film thickness, moisture content, mechanical properties, swelling index, and percentage of drug release at fixed times opted as dependent variables. Results demonstrated a significant effect of all independent variables on the drug release from the prepared bio-composite films. The plasticizer concentration significantly increased the thickness, moisture content, elongation at break, swelling index, and *in vitro* drug release and significantly reduced the tensile strength. The optimized LNZ-loaded bio-composite film comprised of 15% Tween 80 and 30% PEG 400 was highly swellable, elastic, acceptable tensile properties, safe, maintained a moist environment, and indicated great antimicrobial activity against both *Staphylococcus aureus* (ATCC^®^ 25922) and methicillin-resistant *Staphylococcus aureus* (MRSA), which are common wound infectious bacteria. The present study concludes that the optimized LNZ-loaded bio-composite film was successfully designed with fast drug release kinetics and it could be regarded as a promising novel antimicrobial wound dressing formulation.

## Introduction

Wound healing is still an unfulfilled challenge amongst surgical society. Wounds might be lethal in numerous incidents especially those presented by burns, chronic diseases, and post-operative trauma, as they may be readily colonized by microbes and resistant bacteria. Many dressings were developed to cover the surface of the wound as means to generate a proper area for tissue regeneration and perform as a suitable barrier against wound infection (Hafezi et al., 2019; Üstündağ Okur et al., 2019). Wound healing is a complex biological process consisting of healing of both dermal and epidermal tissues *via* their regeneration. It includes a sequence of different phases including, inflammation, migration, proliferation, maturation, and/or remodeling (Colobatiu et al., 2019).

Numerous wound dressings are generally loaded up with antimicrobials to perform against contagious microorganisms and avoid undesirable events such as bacteremia and sepsis. Topical antibiotics play an essential role in treating and preventing abundant dermal bacterial infections due to abrasion, injury, and surgery (Hafezi et al., 2019). Linezolid (LNZ) is a synthetic oxazolidinone antibiotic that has been approved by the Food and Drug Administration (FDA) for the treatment of wound pathogens initiated by Gram-positive bacteria such as methicillin-resistant *Staphylococcus aureus* (MRSA), vancomycin-resistant *Enterococci* (VRE), penicillin-resistant *Streptococcus pneumonia* and glycopeptide intermediate *Staphylococcus aureus* (GISA) (Ma et al., 2015; Hedaya et al., 2017; Carreño et al., 2020). LNZ inhibits the protein synthesis *via* attaching to the 50S ribosomal subunit, therefore obstructing bacterial growth (Haider et al., 2022). As topical wound dressings, LNZ treats various wounds caused by burns and diabetic foot ulcers due to its effectiveness (Ma et al., 2015; Haider et al., 2022).

Nowadays, a wide range of dressing materials including films, hydrogels, hydrocolloids, or foams are applied for diverse types of wounds and target various phases of the wound healing process. Dressing materials such as films are used to treat wounds and deliver therapeutic agents including, antibiotics and anti-inflammatory agents to the wound interface, to manage infection and pain (Colobatiu et al., 2019). Generally, films are biocompatible, biodegradable, and can be easily removed without trauma. They can be applied around different body parts exhibiting different shapes and contours, specifically around joints including knees, elbows, and hips owing to their mechanical properties. When applied over the wound, films promote wound healing by preventing the collection of exudates in the wound bed and maintaining a moist environment at the wound site to enhance tissue regeneration with minimum scar formation (Hafezi et al., 2019).

Bioactive wound dressings are fabricated from biomaterials known to have endogenous activity. Among these biomaterials, there is a vast interest in using natural biopolymers such as chitosan due to its fine film-forming properties, safety, biodegradability, biocompatibility, and affordable price (Li et al., 2020; Sun et al., 2020). Chitosan is also renowned in the wound management field due to its hemostatic, antimicrobial activity, hydrating and anti-inflammatory properties, therefore performing as a wound healing accelerator. One of the effective strategies utilized to improve the properties of chitosan films is by combining chitosan with other biopolymers such as gelatin and Tween 80 to obtain synergistic effects of the constituting components. It turned out to be an interesting approach to formulate new materials with better properties that might not be obtained by only using each individual component (Colobatiu et al., 2019). Gelatin is an inexpensive natural biopolymer derived from collagen through hydrolysis. It is indorsed to be highly biocompatible to human tissues. Gelatin acts as a hemostatic agent to induce wound healing, in addition to high water absorption capacity to absorb exudates existing at the wound bed and hence results in a rapid release profile (Naomi et al., 2021). The non-ionic surfactant, Tween 80, with a hydrophilic-lipophilic balance (HLB) value of 15 is often utilized as an emulsifier, solubilizer, and wetting agent (Jung et al., 2016). It is a safe topical penetration enhancer and an efficient option that has a great impact on wound healing *via* allowing deep drug penetration in wound tissue, hence mounting the interaction between the drug and bacteria cells as well as facilitating efficient topical treatment of infections (Rancan et al., 2021). Compared with the single-component films, bio-composite films of chitosan/gelatin or chitosan/Tween 80 blends have improved physical and mechanical properties. However, their poor flexibility and strong brittleness still greatly limit their application as bioactive wound dressings. Plasticizers are generally incorporated to enhance the elasticity and soften the rigidity of the film structure, as they reduce the inter-molecular forces, create new hydrogen bonds between adjacent polymer chains, enhance the free volume and increase the mobility of the biopolymeric chains. Glycerol and polyethylene glycol 400 (PEG 400) are among those ordinarily used plasticizers owing to their good compatibility and hydrophilic nature that aid bio-films to overcome rigidity and be equipped with the favored flexibility and stretchability (Sun et al., 2020). Thus, it is essential to identify the optimum concentration and type of polymers and plasticizers that can be added to the chitosan-based film formulations with the desired film properties.

This work aimed to develop a novel antimicrobial wound dressing for LNZ-loaded bio-composite films using the solvent casting technique. The I-optimal design was employed to optimize and examine the impact of different types and concentrations of polymers and plasticizers on the thickness, moisture content, mechanical properties, swelling index, and drug release of LNZ-loaded bio-composite films. The main effect and the interaction effects of different variables on thickness, moisture content, mechanical properties, swelling index, and drug release were studied. The optimized bio-composite film formulation flashing promising mechanical properties, *in vitro* drug release, and swelling behavior is expected to accelerate wound healing. Furthermore, the resulting optimized film has been characterized *via* scanning electron microscopy (SEM), Fourier transform infrared spectroscopy (FT-IR), differential scanning calorimetry (DSC), X-ray diffraction (XRD) as well as *in vitro* antibacterial study using disc diffusion test.

## Materials

Linezolid was kindly donated by Pfizer, Egypt. Chitosan (medium molecular weight), Tween 80, and gelatin were all purchased from Sigma-Aldrich Co., USA. Glacial acetic acid, polyethylene glycol 400 (PEG 400), and glycerol were all obtained from Fisher Scientific, U.K. Analytical grade chemicals were used.

## Methods

### Preparation of different bio-composite films

Bio-composite film formulations were prepared according to the solvent casting method adopted by Kan and co-workers (Kan et al., 2019), with slight modifications. To prepare the film-forming solution, chitosan (CS) was dissolved in an aqueous solution of acetic acid 1% volume/volume (v/v) to achieve a concentration of 2% weight/volume (w/v) of chitosan solution. The solution was continuously stirred overnight at room temperature at 500 rpm to guarantee complete dissolution of chitosan. A fixed amount of linezolid (LNZ) (50 mg) was dissolved in the previously prepared solution. Either polyethylene glycol 400 (PEG 400) or glycerol (G) were then added as plasticizers under gentle stirring at 40 °C for 1 h to obtain a good blend. In case of chitosan/Tween 80 (CS/T80) or chitosan/gelatin (CS/Gel) films, weighed amount of Tween 80 or gelatin was dissolved in the chitosan solution. The composition of the prepared bio-composite film formulations is listed in Table 1. The obtained filmogenic solutions were degassed under a vacuum for 45 minutes to eliminate all trapped air bubbles. Afterward, solutions were cast into glass Petri dishes (9 cm^2^) and dried in an electric oven at 40 °C for 48 h. All dried films were peeled off manually from the casting surface, conditioned at 25 ± 0.2 °C, 50 ± 5% relative humidity for 48 h, and cut into small pieces of 3 cm^2^ for further analysis. The bio-composite films were prepared in triplicates.

**Table 1.** Formulations codes of the LNZ-loaded bio-composite films.

Ingredients (%)	Formulations codes														
	F1	F2	F3	F4	F5	F6	F7	F8	F9	F10	F11	F12	F13	F14	F15
Gelatin	–	–	–	–	–	–	–	–	–	–	2	2	2	2	2
Tween 80	15	15	15	15	15	–	–	–	–	–	–	–	–	–	–
Glycerol	–	15	30	–	–	–	15	30	–	–	–	15	30	–	–
PEG 400^a^	–	–	–	15	30	–	–	–	15	30	–	–	–	15	30

*All bio-composite film formulations contain 2% (w/v) chitosan, as the film forming material, and fixed amount of LNZ (50 mg).

^a^PEG 400 indicates polyethylene glycol 400.

### Weight variation

The weight of the formulated bio-composite films was determined *via* cutting each film sample into small pieces (3 cm^2^ area). The weight of each sample was accurately measured using a digital electronic weighing balance (ADAM, Model PW 124, UK). The test was carried out in triplicates for each film formulation. Mean and standard deviation were calculated.

### Film thickness

The film thickness was achieved according to the method of Sun et al. and measured using a hand-held digital micrometer (Freemans, Model ID-FDOM25, India), with 0.001 mm sensitivity (Sun et al., 2020). Each film sample was measured at six random points including the center. The mean thickness and standard deviation were calculated for each film sample.

### Moisture content

The moisture content of bio-composite films was studied by primarily weighing each film formulation separately using a digital electronic weighing balance. The films were dried in an electrical oven (JOUAN, Model EU 18, France) at 105 °C for 24 h until equilibrium weight was obtained. Dried films were re-weighed and the weight loss of each film was recorded, from which the percentage moisture content was estimated using the equation below.

(1) $$\text{Moisture content }(\%)\;=\;\frac{(M_{i}\;-\;M_{f})\;}{M_{i}}\;\times \;100$$

*M_i_ = mass of the initial sample *M_f_ = mass of the dried sample

The test was performed in triplicates for each film formulation. Mean and standard deviation were calculated.

### Determination of surface pH

The surface pH of the formulated bio-composite films was measured by adopting the method of Ghosal and co-workers (Ghosal et al., 2019). Each film sample was cut into small pieces and completely dissolved in 15 mL of distilled water with the aid of a magnetic stirrer. JENWAY pH meter (Model 3510, UK) was used for measuring the surface pH. Three readings were taken for each film sample. Mean and standard deviation were calculated.

### Folding endurance

The folding endurance was studied by manually folding the formulated film samples repetitively in the same place till they crack. The number of times the film sample can be folded at the same point without cracking presented the value of the folding endurance. Film samples that can get folded ≥ 300 times without cracking were supposed to pass this test (Bassi & Kaur, 2017).

### Mechanical properties

The mechanical properties of the tensile strength (TS) and elongation at break percent (EB) of the film formulations were studied using an INSTRON 3356 Texture Analyzer, UK. The initial grip separation and the mechanical crosshead speed were adjusted at 32 mm and 10 mm/minute, respectively, and equipped with a 1 kN static load cell (Francois & Debandi, 2016). Film formulations were cut into small strips, held between two clamps, and pulled by the top clamp. Hence, the TS and EB were determined once the film sample broke off. Mechanical tests were conducted in triplicate. Mean and standard deviation were reported for each film formulation.

### Swelling studies

The swelling ability of the formulated bio-composite films was studied using the method adopted by Hafezi and co-workers (Hafezi et al., 2019). All prepared dry film formulations were cut into small pieces and weighed carefully to get an accurate weight. The films were then immersed in phosphate buffer saline (PBS) (pH 7.4) at 37 ± 1 °C for 24 h. At regular time intervals, swollen film samples were taken out and wiped gently using a filter paper to get rid of the adsorbed solution, then weighed instantly on a digital balance. The swelling index (SI) was determined using the equation below.

(2) $$\text{SI}(\%) =\frac{\left({\;W}_{s}\;-\left.W_{d}\right)\;\right.}{W_{d}}\mathrm{\times 100}$$

Where, *W_s_ and W_d _*represent the weights of swollen film sample and initial dry film sample, respectively. The test was performed in triplicate. Mean and standard deviation were estimated for each film sample.

### Drug content determination

Each film sample was cut into small pieces from five different regions of the film including the center. Each film sample was placed in 10 mL distilled water at 37 ± 0.2 °C and stirred overnight using a magnetic stirrer until completely dissolved (Pawar et al., 2013). The obtained solutions were then diluted with distilled water and assayed using a UV spectrophotometer (SHIMADZU Corporation, Model UV-1800 240 V, Japan) at a fixed λ_max_ value of 251 nm against distilled water as a blank. The percentages of the drug content were calculated using the following equation:

(3) $$\text{Drug content}(\%) =\frac{AQ}{TQ}\mathrm{\times 100}$$

*AQ is the actual quantity of the drug available in the film. *TQ is the theoretical quantity of the drug.

The experiment was repeated in triplicates. The mean and standard deviation were calculated for each film sample.

### In vitro drug release studies

*In vitro* release studies of LNZ from bio-composite films and free LNZ were conducted using the Incubator Shaker apparatus (ZHICHENG ZHWY-2102C, China). Working conditions of the equipment were adjusted to incubate at 32 ± 0.2 °C to mimic the skin temperature and at a rotational speed of 50 rpm throughout the experiment (Jantrawut *et al.*, 2017). Each film sample of 3 cm^2^ was cut and immersed in a glass vial comprising 100 mL of PBS (pH 7.4) as a dissolution medium. Aliquots of 3 mL were withdrawn using a syringe from each glass vial at fixed time intervals at 5, 10, 20, 30, 45, 60, 90, 120 minutes, then at 3, 4, 6, and 8 h, and finally at 24 h. All of the withdrawn aliquots were immediately replenished with an equivalent volume of fresh PBS (pH 7.4) after each sampling to maintain a constant medium volume throughout the experiment. The content of linezolid released from each film sample was determined spectrophotometrically using a UV spectrophotometer (SHIMADZU Corporation, Model UV-1800 240 V, Japan) at a wavelength of 251 nm. *In vitro* release studies were conducted in triplicate and were expressed as a cumulative percentage of drug release *versus* time.

### Design of experiments

The design of experiments using response surface methodology (RSM) was employed to study the effect of independent variables on a range of responses (dependent variables). Polymer concentration (X_1_), plasticizer concentration (X_2_), polymer type (X_3_), and plasticizer type (X_4_) were chosen as the independent variables. Whereas, the film thickness (Y_1_), moisture content (Y_2_), tensile strength (Y_3_), elongation at break (Y_4_), swelling index (Y_5_), and the percentage drug release at 30 and 180 minutes (Y_6_ and Y_7_, respectively) were chosen as the dependent variables. The experimental range and levels of the independent variables are presented in Table 2. I-optimal design using Design-Expert^®^ version 12.0.3.0 software (Stat-Ease Inc., USA) was used for experimental planning and statistical analysis. The design proposed a total number of 19 sets of experimental runs, with 4 center points as replicates for the estimation of the experimental error. Randomized order of the experimental runs was used to reduce the effect of uncontrolled factors. Table 3 displays the experimental runs of the design matrix used to assess the impact of independent variables and the experimental values of the measured responses for different LNZ-loaded bio-composite films. The influence of independent variables was examined by I-optimal design under RSM using the following equation:

(4) $${Y=\beta }_{0}{+\beta }_{1}X_{1}{+\beta }_{2}X_{2}{+\beta }_{3}X_{3}{+\beta }_{4}X_{4}{+\beta }_{12}X_{1}X_{2}{+\beta }_{13}X_{1}X_{3}{+\beta }_{14}X_{1}X_{4}{+\beta }_{23}X_{2}X_{3}{+\beta }_{24}X_{2}X_{4}{+\beta }_{34}X_{3}X_{4}$$

Where, Y is the response, while the constant β_0_ is the intercept coefficient of the 19 runs. The terms β_1_, β_2_, β_3_, and β_4_ represent the single variable (linear coefficient); the interaction terms β_12_, β_13_, β_14_, β_23_, β_24_, and β_34_ indicate the extent of interactions between variables (second order interaction coefficient).

**Table 2.** Experimental range and coded levels of the independent variables for I-optimal design.

Independent variables	Levels of variables		
	–1^a^	0^a^	+1^a^
X_1_: Polymer concentration (%)	0	2	15
X_2_: Plasticizer concentration (%)	0	15	30
X_3_: Polymer type	Gelatin	Tween 80	–
X_4_: Platicizer type	Glycerol	PEG 400^b^	–

**^a^**(+1), (0), and (–1) codes express the high, basal, and low levels of the statistical experiment matrix, respectively. **^b^**PEG 400 indicates polyethylene glycol 400.

**Table 3.** Evaluation parameters and formulations of I-optimal design matrix and their experimental values of responses for the LNZ-loaded bio-composite films.

Run	X_1_: Polymer concentration (%)	X_2_: Plasticizer concentration (%)	X_3_: Polymer type	X_4_: Platicizer type	Y_1_ (mm)	Y_2_ (%)	Y_3_ (MPa)	Y_4_ (%)	Y_5_ (%)^a^	Y_6_ (%)	Y_7_ (%)	Weight variation (g)	Surface pH	DC (%)^b^
1	15	15	Tween 80	Glycerol	0.202	18.89	18.93	61.11	2004.44	60.92	85.28	0.045	5.40	99.52
2	0	30	Tween 80	PEG 400	0.159	13.75	6.01	78.46	2010.63	65.31	87.77	0.040	5.45	99.27
3	2	30	Gelatin	PEG 400	0.170	15.66	6.13	70.17	2022.89	60.35	82.85	0.042	5.50	99.70
4	15	30	Tween 80	PEG 400	0.206	19.59	5.19	88.18	2047.42	71.92	93.42	0.048	5.48	99.86
5	15	15	Tween 80	PEG 400	0.146	14.29	16.42	79.74	1845.45	67.14	91.54	0.038	5.42	99.77
6	2	0	Gelatin	Glycerol	0.099	10.91	61.71	8.24	1537.27	42.81	68.61	0.028	5.40	99.17
7	15	0	Tween 80	Glycerol	0.125	13.73	53.00	21.38	1615.64	57.34	82.55	0.033	5.40	99.56
8	2	15	Gelatin	PEG 400	0.114	11.77	18.03	60.22	1778.68	56.72	79.26	0.034	5.48	99.64
9	0	0	Gelatin	Glycerol	0.088	10.21	56.92	12.67	1309.18	50.45	73.97	0.025	5.40	98.94
10	0	30	Tween 80	Glycerol	0.184	17.35	10.52	45.73	2077.55	57.63	81.62	0.049	5.50	99.40
11	0	15	Tween 80	Glycerol	0.122	12.86	20.12	57.32	1903.57	54.41	79.02	0.035	5.47	99.93
12	2	0	Gelatin	Glycerol	0.096	10.55	61.32	9.40	1539.29	42.81	68.53	0.029	5.44	99.17
13	15	0	Tween 80	Glycerol	0.128	13.43	51.32	20.12	1611.94	57.43	81.64	0.034	5.41	99.55
14	2	15	Gelatin	Glycerol	0.125	13.16	22.17	49.67	1931.58	46.90	71.58	0.038	5.46	99.92
15	0	15	Tween 80	Glycerol	0.123	12.00	21.18	59.87	1905.06	54.34	79.00	0.037	5.44	99.92
16	0	15	Gelatin	PEG 400	0.095	10.45	17.26	69.75	1659.70	62.43	84.01	0.034	5.42	99.24
17	2	30	Gelatin	Glycerol	0.211	20.76	14.75	41.94	2140.57	50.74	74.25	0.053	5.49	99.14
18	2	30	Gelatin	PEG 400	0.166	16.14	6.41	69.56	2023.90	60.29	82.73	0.043	5.47	99.71
19	15	30	Tween 80	Glycerol	0.239	24.40	9.11	57.67	2195.12	63.84	88.83	0.062	5.44	99.22

Note: Results are mean values of three replicates and the overall standard deviation values were < ±5.5.

^a^Swelling index values after 20 minutes. **^b^**DC: Drug content.

Appropriate models for I-optimal design involve linear and 2FI (two factorial) models. The obtained regression models for each response were validated by analysis of variance (ANOVA) in the software and *p*-value. Besides, significant terms were used to assess the reliability of the selected model. Typically, a regression model with a good fit should have a high determination coefficient (R^2^) value. Adjusted and predicted R^2^ should be in reasonable agreement for the model to be fit, *i.e.* difference between them < 0.2. Predicted residual sum of square (PRESS) value > 4 verifies the reproducibility of the model. An adequate precision ratio > 4 is desirable and confirms that the model can be utilized to navigate the design space. *p*-value < 0.05 combined with a high *F*-value determines the factor that has the greatest significant effect on the response (Issa *et al.*, 2020).

### Scanning electron microscopy (SEM)

The surface morphology of optimized LNZ-loaded bio-composite film was evaluated using a scanning electron microscope (JEOL-JSM 6380 LV, Japan) set at 15 kV accelerating voltage. Images of the film sample were acquired at different magnifications (Li et al., 2020).

### Fourier-transform infrared spectroscopy (FT-IR)

FT-IR spectra of the optimized LNZ-loaded bio-composite film and its individual components were determined by using a SHIMADZU IRAffinity-1S spectrometer (SHIMADZU Corporation, Japan), operating for the wavenumber range from 4000 to 400 cm^−1^, with a resolution of 2 cm^−1^ by accumulating 24 scans (Ediyilyam et al., 2021). The FT-IR spectrometer was equipped with LabSolutions IR software (SHIMADZU Corporation, Japan) and a diamond universal attenuated total reflection (ATR) unit.

### Differential scanning calorimetry (DSC)

DSC thermograms of optimized LNZ-loaded bio-composite film and its individual components were obtained using a Setaram LABSYS evo instrument, France. Samples (5 mg) were hermetically sealed in aluminum pans and heated from 25 to 300 °C at the rate of 10 °C.min^−1^ under a nitrogen atmosphere with a flow rate of 50 mL.min^−1^ (Ghosal *et al.*, 2019). An empty aluminum pan was employed as a reference. Experiments were conducted in triplicate.

### X-ray diffraction (XRD)

XRD patterns of optimized LNZ-loaded bio-composite film and its pure starting materials (chitosan and LNZ) were achieved using a Bruker D8 DISCOVER X-ray diffractometer, Germany. Samples were conducted with Ni-filtered Cu K*_α_* radiation (*λ* = 0.15416 nm), under a voltage of 45 kV and current of 40 mA. The rate of scanning was set at 2° min^−1^ over a diffraction angle (2*θ*) ranging from 5° to 100° (Zhang et al., 2020).

### In vitro antibacterial activity by disc diffusion method

The antibacterial activity of bio-composite films was examined *via* a disc diffusion test according to the clinical and laboratory standards institute (CLSI) guidelines with modifications (Boncu et al., 2020). The test was performed using one of the common wound infection pathogens such as *Staphylococcus aureus* (ATCC^®^ 25922) and a challenging resistant strain of methicillin-resistant *Staphylococcus aureus* (MRSA) isolated in a previous study from dental clinic surface (Ma et al., 2015; Hedaya et al., 2017; Carreño et al., 2020). Pure bacterial colonies from an overnight culture of *Staphylococcus aureus* and MRSA strain were suspended each in sterile saline and turbidity was adjusted to be equivalent to 0.5 McFarland. The bacterial suspension was spread on the Mueller Hinton agar (Oxoid, UK) surface using a sterile cotton swab. Then, the plates were left to dry for 5 minutes. Control and optimized film were cut into small discs of diameter 6 mm, equivalent to the size of the reference antibiotic disc and containing an equivalent amount of LNZ (30 μg) in the antibiotic disc. A space of 20 mm was kept from the plates’ edges to prevent the intersection of the zones of inhibition (ZOI). The plates were then incubated at 37 °C for 24 h. The antibacterial effect was studied by measuring the diameters of the ZOI. All studies were performed in duplicate. ZOI of diameter ≥21 mm was interpreted as susceptible according to the CLSI guideline (30^th^ Edition, 2020).

### Sterility test for bio-composite films

Test samples were prepared by dissolving a 6 mm disc of each of the control and optimized bio-composite film into 9 mL PBS (pH 7.4). Afterward, 1 mL of the solution was transferred into 9 mL casein soya bean digest broth. The medium was incubated for 48 h at 37 °C ± 0.2. After incubation, the medium was checked for growth (Ahmed et al., 2018).

## Results and discussion

### Preparation of different bio-composite films

Solvent casting method is a widely used approach in the preparation of film dressings for wound healing applications. It has been the leading manufacturing technique for marketed films due to the ease of manufacturing and cost-effectiveness. Some essential key parameters should be presented for the success of the solvent casting method. These key elements involve three main perks including, the selection of polymers that are soluble in the potential solvent used, the film-forming solution has to exhibit sufficient solid content, and finally, the prepared film has to be uniform and easily peeled from the casting surface (Shamma & Elkasabgy, 2016). In our solvent casting studies, chitosan was chosen as the film polymer, thus fulfilling all the required perks. The presence of hydroxyl (–OH) and amino (–NH_2_) groups excels chitosan with outstanding film-forming competency throughout non-covalent interactions (Ma et al., 2017). Either PEG 400 or glycerol was incorporated as a plasticizer to modify the film properties.

**Figure 1.**
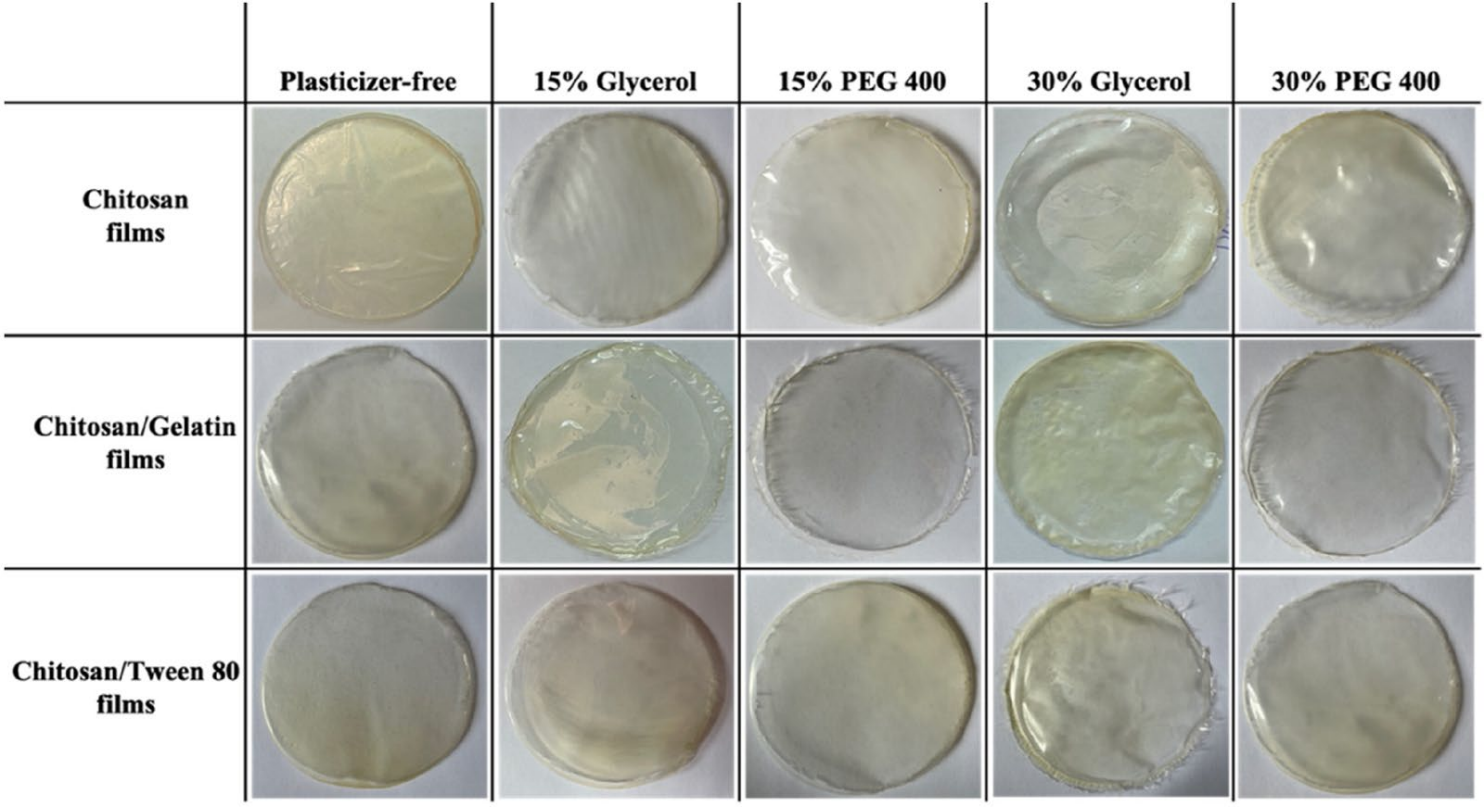
Pictures of LNZ-loaded bio-composite films using different plasticizer types and concentrations.

Photographs of the formulated bio-composite films using different plasticizer types and concentrations are shown in Figure 1. Visual observations indicated that the prepared films were uniform, transparent, translucent, and continuous without any cracks or bubbles. Transparency is an essential property for film-based wound dressings, as it permits the inspection of the wound bed without the necessity to remove the wound dressing (Boateng et al., 2013). Also, it should be noted that a suitable amount of plasticizer provided a smoother, more flexible, and homogenous surface for the films, thus they can be easily peeled and handled from the casting surface (Sanyang et al., 2016). This is attributed to the plasticization effect that destroys the tight ordered network structure of chitosan and forms new hydrogen bonds (Sun et al., 2020). The addition of LNZ drug molecules conveyed a slight translucence to the films, nevertheless, they remained fairly transparent (Boateng et al., 2013). Film color is a chief aspect in terms of consumer acceptance. Hence, it was observed that the obtained bio-films exhibit a yellowish coloration due to the presence of chitosan (Lamim et al., 2006). In our research, numerous parameters such as weight variation, thickness, moisture content, surface pH, folding endurance, mechanical properties, swelling studies, drug content, and *in vitro* drug release studies were conducted to evaluate the potential use of the formulated bio-composite films for wound healing.

### Weight variation

The weight variation of 3 cm^2^ films, measured using a digital electronic weighing balance, ranged from 0.025 ± 0.001 to 0.062 ± 0.001 g, as displayed in Table 3. Plasticizer-free films witnessed the least weight in comparison to that plasticized ones. Results revealed that increasing the polymer and the plasticizer contents resulted in heavier films.

### Statistical analysis of I-optimal experimental design

I-optimal experimental design was conducted to demonstrate the response surface nature in the experimental runs and to detect the optimal LNZ-loaded bio-composite film. The design built up mathematical equations to correlate and identify the influence of the formulation variables of X_1_ (polymer concentration), X_2_ (plasticizer concentration), X_3_ (polymer type), and X_4_ (plasticizer type) on each response of the bio-composite films (Ahmed et al., 2018). Table 4 displays the outcome of regression for the measured responses. Estimation of the response values for thickness (Y_1_), tensile strength (Y_3_), elongation at break (Y_4_), and swelling index (Y_5_) in virtue of the linear model was the most fitting, whereas the 2FI model was selected to estimate the response values of the moisture content (Y_2_) and percentage drug release after 30 and 180 minutes (Y_6_ and Y_7_, respectively). This is attributed to the high values for R^2^ that indicate that the statistical model can elucidate the variability in the responses. Additionally, the reasonable agreement between adjusted and predicted R^2^ with a difference of < 0.2 between their values results in a reliable model (Table 4). Besides, the model of each response was significant with a *p*-value < 0.05 and a high model *F*-value (Table 4). Adequate precision showed values > 4, which is desirable. Thus, this model can be employed to navigate the design space.

**Table 4.** Summary of the regression outcome for the measured responses.

Source of variance	Responses						
	Y_1_	Y_2_	Y_3_	Y_4_	Y_5_	Y_6_	Y_7_
Model	Linear	2FI^a^	Linear	Linear	Linear	2FI^a^	2FI^a^
*p*-value	< 0.0001	< 0.0001	< 0.0001	< 0.0001	< 0.0001	< 0.0001	< 0.0001
F-value	99.23	27.14	34.52	18.87	40.20	582.29	350.36
SD^b^	0.0094	0.8656	7.00	11.26	77.74	0.4362	0.5098
R^2^	0.9659	0.9784	0.9079	0.8436	0.9199	0.9986	0.9977
Adjusted R^2^	0.9562	0.9514	0.8816	0.7989	0.8970	0.9969	0.9949
Predicted R^2^	0.9416	NA^c^	0.8381	0.7174	0.8623	NA^c^	NA^c^
Adequate precision	33.3266	22.0567	15.5824	12.5707	18.3474	85.9358	65.6615
PRESS^d^	0.0021	NA^c^	1205.86	3206.31	1.455E05	NA^c^	NA^c^

^a^2FI: two factorial interaction model.

^b^SD: standard deviation.

^c^Cases with leverage of 1: Predicted R^2^ and PRESS statistics are not defined.

^d^PRESS: predicted residual sum of square.

### Thickness

**Figure 2.**
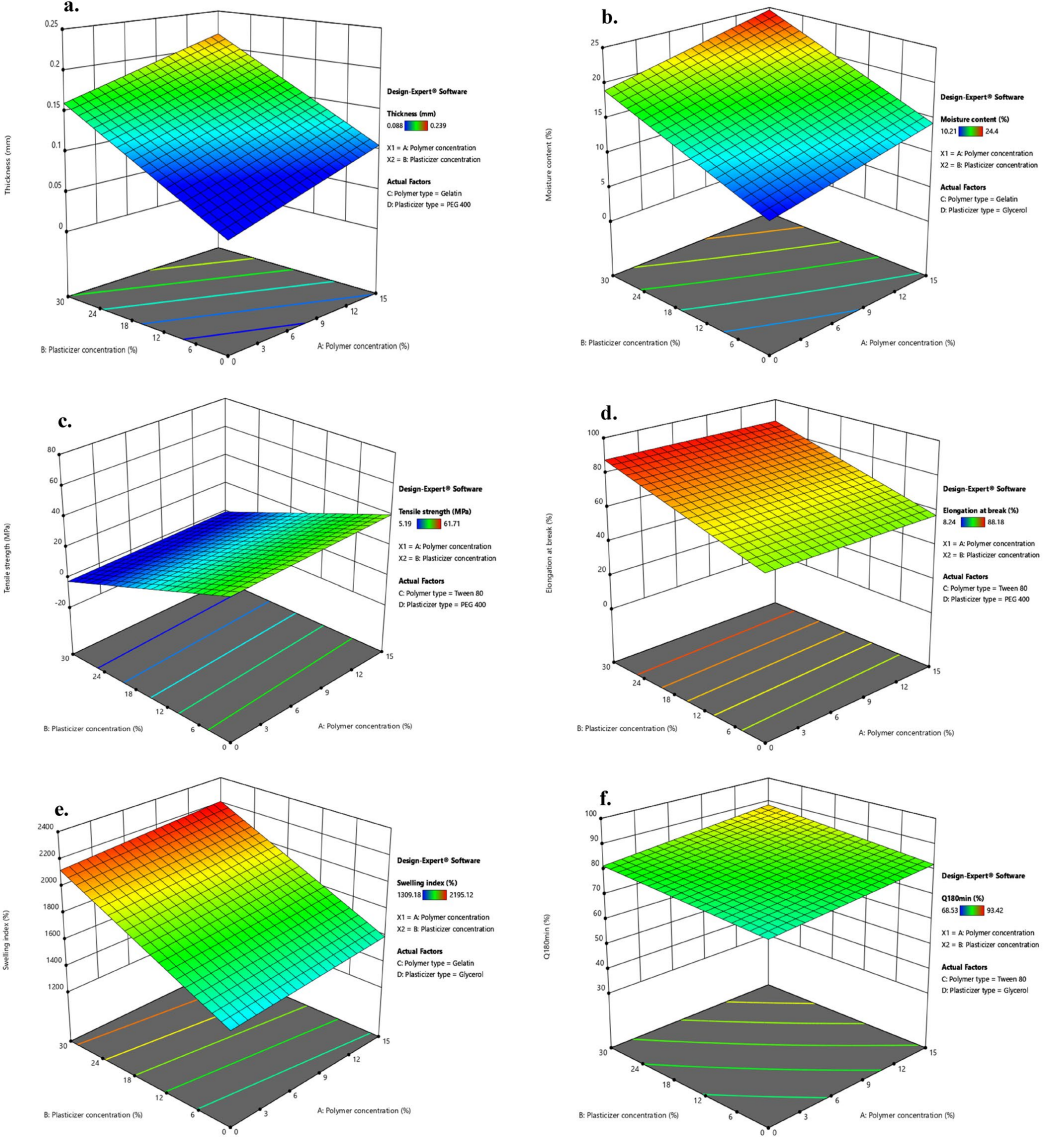
Response surface plot of the impact of polymer concentration (X_1_) and plasticizer concentration (X_2_) on the a. thickness, b. moisture content, c. tensile strength, d. elongation at break, e. swelling index of LNZ bio-composite films, and f. percentage of LNZ release after 180 minutes.

The thickness of different LNZ-loaded bio-composite films ranged from 0.088 ± 0.01 − 0.239 ± 0.02 mm. The films thickness is acceptable and can be called stretch films, as the American Society for Testing Materials (ASTM) precisely outlines ‘film’ as having a nominal thickness of *≤* 0.250 mm (Singh *et al.*, 2021). Statistical analysis using ANOVA was employed to assess the level of significance of the tested factors on the thickness of different LNZ-loaded bio-composite films. Figure 2(a) displays the response surface plot of the impact of polymer concentration (X_1_) and plasticizer concentration (X_2_) on the thickness of LNZ-loaded bio-composite films. Results showed that both the polymer concentration (X_1_) and the plasticizer concentration (X_2_) had significant effects on the film thickness (*p*-value < 0.0001 for both variables). Rising the polymer concentration caused a significant increase in the film thickness. This can be ascribed to the dilation in the chitosan polymer backbone upon cross-linking (Ibrahim *et al.*, 2020). As the polymer concentration increases in the film-forming solution, it increases the dry matter content resulting in thicker films. Similar results for the impact of polymer concentration on the film thickness were found in the literature (Ibrahim et al., 2020; Singh et al., 2021). A significant effect of the plasticizer concentration on the film thickness was also determined. Rising the plasticizer concentration from 0% to 30% caused a significant increase in the film thickness. This can be endorsed by the role of plasticizers in disrupting and restructuring inter-molecular polymer chain networks of chitosan, thus generating more free volumes (Sanyang et al., 2016). Similar impact of the plasticizer concentration on the film thickness was reported by Ibrahim and co-workers and Sun and co-workers (Ibrahim et al., 2020; Sun et al., 2020).

**Figure 3.**
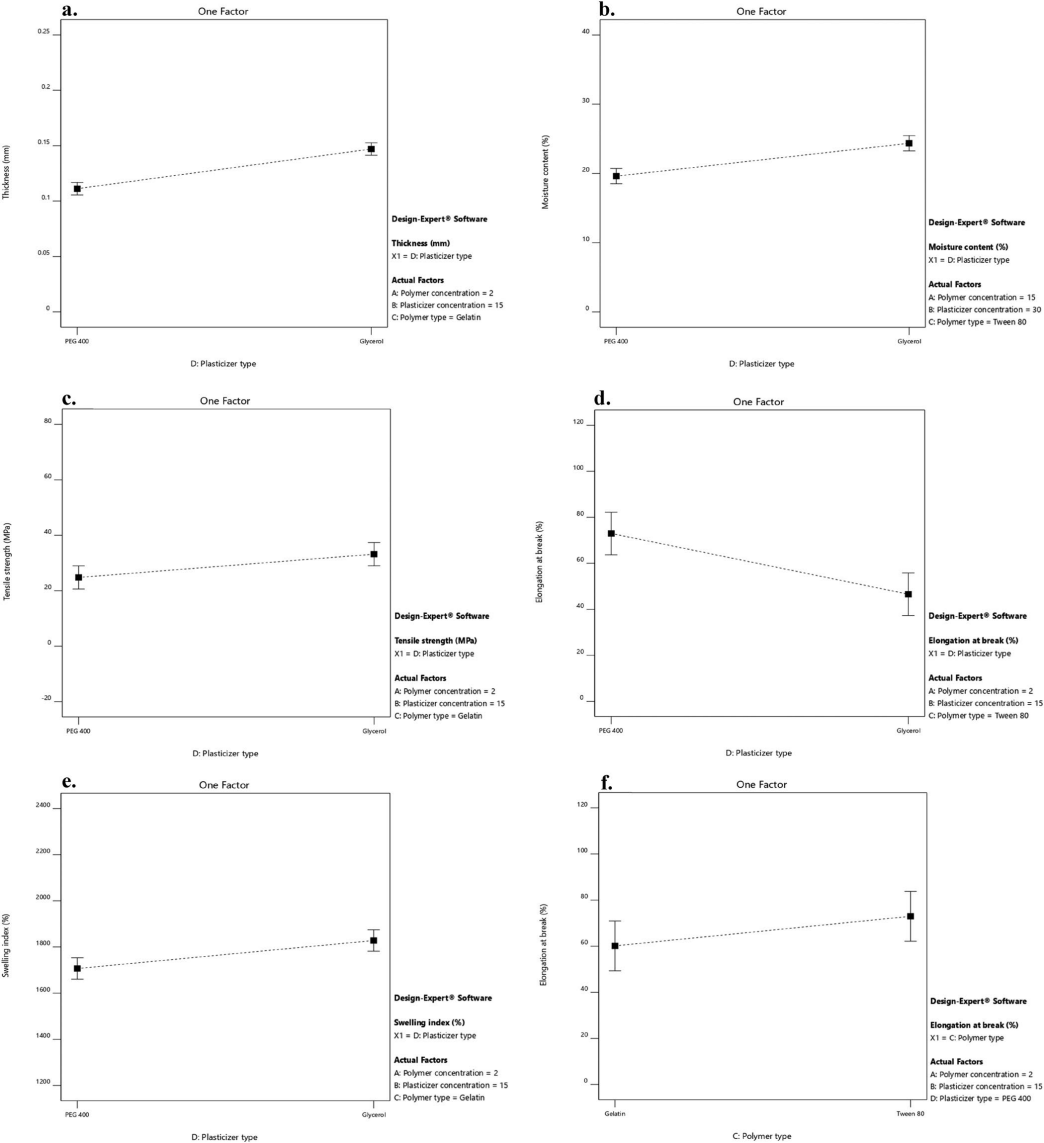
Impact of the plasticizer type (X_4_) on the a. thickness, b. moisture content, c. tensile strength, d. elongation at break, and e. swelling index and the effect of the polymer type (X_3_) on the f. elongation at break of LNZ bio-composite films.

Figure 3(a) displays the impact of the plasticizer type on the thickness of LNZ-loaded bio-composite films. A significant effect (*p*-value < 0.0001) of the plasticizer type on the film thickness was demonstrated. The impact of the plasticizer type on increasing the film thickness was more pronounced for glycerol than PEG 400. Glycerol molecules tend to expand the spacing between the chitosan macromolecules located in each layer rather than separating them and interact by hydrogen bonds at the chitosan-specific sites (–OH and –NH_2_) (Francois & Debandi, 2016). Taking into account the hygroscopic nature of glycerol, the ability to absorb ambient moisture between its molecules resulted in thicker films (Manshor et al., 2018). As was reported by Ghasemlou et al. and Sun et al., glycerol can penetrate the polymer network easily and rapidly, resulting in a thicker film (Ghasemlou et al., 2011; Sun et al., 2020).

### Moisture content

The experimental results support that the differences in film thickness can possibly contribute to the differences in moisture content of the bio-composite films as a strong positive correlation was exhibited between those two parameters. ANOVA test was carried out to assess significant terms of the tested factor on the moisture content. Figure 2(b) displays the response surface plot of the impact of polymer concentration (X_1_) and plasticizer concentration (X_2_) on the moisture content of LNZ-loaded bio-composite films. The results display that only the plasticizer concentration (X_2_) showed a significant impact on the moisture content (*p*-value < 0.0001). Rising the plasticizer concentration from 0% to 30% caused a significant increase in the moisture content. This tendency can be rationalized by the physical interactions between the plasticizer and chitosan enabling water to be entrapped within the polymer skeleton resulting in structural modifications of the polymer network (Ibrahim et al., 2020). The network as a result becomes less dense because of the increase in the mobility of the polymeric chains. Hence, these consequences of the plasticizing effect are favorable for the adsorption or desorption of water molecules (Song & Tang, 2011). Numerous studies reported that the moisture content of bio-composite films increases by adding more plasticizer (Rivero et al., 2016; Ibrahim et al., 2020).

A significant effect of the plasticizer type on the moisture content was determined (*p*-value = 0.0002). It is clear that glycerol-plasticized films showed higher moisture content than PEG 400-plasticized films (Figure 3(b)). This may be possibly attributed to the strong hydrophilic nature of glycerol, which is favorable to retain water molecules in the film matrix (Manshor et al., 2018). Glycerol has three primary hydroxyl groups, which generated the most contribution to the water adsorption compared with PEG 400 which has two primary hydroxyl groups (Sun et al., 2020). Hence, glycerol acts as a water-holding agent and has a strong affinity to water molecules enabling it to easily develop hydrogen bonds (Sanyang et al., 2016). Similar results were obtained by Bajdik et al. for films prepared with chitosan and ascorbic acid and plasticized with glycerol and PEG 400 (Bajdik et al., 2009).

### Determination of surface pH

The surface pH of the bio-composite films was slightly acidic in the range between pH 5.40 ± 0.01 – pH 5.50 ± 0.01 in distilled water, as shown in Table 3. The estimated results are following the average range of the natural skin surface pH. This presumably ensures that the bio-composite films will be non-irritant to the skin. The obtained results are consistent with the observations of Ibrahim et al. for chitosan/citrate films and Ghosal et al. for polymeric networks-based bio-composite alginate films (Ghosal et al., 2019; Ibrahim et al., 2020).

### Folding endurance

Folding endurance is the resistance of the film samples to crack upon folding (Zaman & Hanif, 2018). The bio-composite film samples passed the folding endurance test except for the plasticizer-free films. This indicates that plasticization improved the folding endurance of the film samples *via* providing better elasticity and pliability to the films and hence can attach to the skin without cracking. Similar findings were reported by other authors (Velayutham & Manivannan, 2015; Ghosal et al., 2019).

### Mechanical properties

An ideal wound dressing should be flexible in order to be compliant with the skin morphology and movements. It should as well possess adequate resistance to retain the integrity and withstand mechanical stress during processing, storage, usage, and peeling off (Ediyilyam et al., 2021). The results in Table 3 showed that plasticized films had a TS in the 5.19 ± 2.89 − 22.17 ± 4.49 MPa range and an EB in the 41.94 ± 2.44 − 88.18 ± 3.02% range. As reported in the literature, normal skin possesses TS between 2.5 − 16 MPa and EB of approximately 70% (Ma et al., 2017). Accordingly, plasticized bio-composite films exhibit a typical elastomeric behavior and are suitable for potential application as wound dressings (Francois & Debandi, 2016).

### Tensile strength

Statistical analysis using ANOVA was employed to assess the level of significance of the tested factors on the TS of various LNZ-loaded bio-composite films. Figure 2(c) displays the response surface plot of the impact of polymer concentration (X_1_) and plasticizer concentration (X_2_) on the TS of LNZ-loaded bio-composite films. The results demonstrate that only the plasticizer concentration (X_2_) had a significant impact on the TS (*p*-value < 0.0001). Rising the plasticizer concentration from 0% to 30% caused a significant decrease in TS. An inversely proportional relationship between plasticizer concentration and TS can be elucidated by the dominance of the strong hydrogen bonding generated by polymer–plasticizer interaction over polymer–polymer inter-molecular attraction (Proaño et al., 2020). Thus, allowing more sliding chains as well as increasing the free volume (Sun et al., 2020). Besides, increasing the plasticizer concentration leads to a significant increase in the moisture content of the films due to the high hygroscopic and humectant nature of plasticizers, which also interfere with the interactions between the polymeric chains of adjacent macromolecules and hinder the TS (Cerqueira et al., 2012). Numerous investigations reported that the TS of films decreases by incorporating more plasticizer (Hafezi et al., 2019; Sun et al., 2020).

Figure 3(c) displays the impact of the plasticizer type on the TS of LNZ-loaded bio-composite films. A significant effect of the plasticizer type on the TS was validated (*p*-value = 0.0490). The effect of the plasticizer type on decreasing the TS was more pronounced for PEG 400 than glycerol. This suggests that PEG 400 could be a better softener with enhanced efficiency for plasticizing the bio-composite films. The effectiveness of PEG 400 is most likely due to that it acts sustainably more as an external plasticizer. As it stays mobile in the polymeric film matrix to soften the rigid structure of the film and boosts the mobility of the acetamide groups of chitosan (Domján et al., 2009). Also, an interesting finding that is worth mentioning is the influence of the plasticizer molecular weight on the plasticization efficiency (*i.e.* decreasing TS). The plasticizer efficiency boosts upon increasing the molecular weight. Accordingly, PEG 400 (400 g/mol) has a higher molecular weight than glycerol (92 g/mol) (Suyatma et al., 2005). Other researchers have also reported a similar observation with other polysaccharide films where PEG 400 showed greater plasticizing efficiency as compared to other polyols (Hafezi et al., 2019; Sun et al., 2020).

### Elongation at break

The influence of the plasticizer concentration on the EB of bio-composite films has illustrated an inverse behavior in comparison to its correspondent TS. ANOVA test was employed to assess significant terms of the tested factor on the EB. Figure 2(d) displays the response surface plot of the impact of polymer concentration (X_1_) and plasticizer concentration (X_2_) on the EB of LNZ-loaded bio-composite films. The results display that only the plasticizer concentration (X_2_) had a significant influence on the EB (*p*-value = 0.0022). Rising the plasticizer concentration from 0% to 30% caused a significant increase in the EB. This could be ascribed to the capacity of plasticizers to improve the biopolymers flexibility by decreasing the glass transition temperature and enlarging the interspaces between the chitosan polymer chains (Hafezi et al., 2019). Given the fact that plasticization can possibly overcome the brittle behavior of chitosan films, resulting in more ductile films (Üstündağ Okur et al., 2019). Similar EB behavior has been reported for other polysaccharide films (Zaman & Hanif, 2018; Sun et al., 2020).

Figure 3 shows the impact of the independent variables of polymer type (X_3_) and plasticizer type (X_4_) on the EB of LNZ-loaded bio-composite films. Results display that both factors had a significant effect on the EB (*p*-value = 0.0415 for X_3_ and *p*-value = 0.0005 for X_4_). The effect of the polymer type on increasing the EB was less pronounced for gelatin than Tween 80 (Figure 3f). This can be anticipated by the addition of gelatin which results in much stiffer films due to increasing the crystallinity (Kan et al., 2019). The cross-linking between chitosan chains and gelatin protein chains *via* electrostatic and non-covalent interactions results in the development of a stable and dense 3D network that impact the EB (Colobatiu et al., 2019). Furthermore, upon drying the films, considerable water loss occurs that might lead to the disruption of the isotropic gel structure of gelatin and squashing its crystallites (Arvanitoyannis et al., 1998). Similar findings were reported by Üstündağ Okur and colleagues where drug-loaded chitosan films showed better mechanical properties than drug-loaded chitosan/carbopol films (Üstündağ Okur et al., 2019).

A significant effect of the plasticizer type on the EB was determined. The effect of the plasticizer type on increasing the EB was less pronounced for glycerol than PEG 400 (Figure 3d). This can be elucidated by the fact that glycerol acts considerably more as an internal plasticizer by being immobilized in the film matrix. This results in reducing the mobility of the chitosan acetamide groups and the formation of non-covalent bonds between polymer chains (Domján et al., 2009). Additionally, the anti-plasticization effect or phase separation phenomenon takes place in highly plasticized films with glycerol (*i.e.* 30%) (Table 3). This results in the formation of glycerol-rich and chitosan-rich moieties due to the migration of glycerol from the film matrix, enabling adjacent chitosan molecules to strongly interact once more and decrease the EB (Conzatti et al., 2018). This tendency has been reported in other authors’ previous work (Üstündağ Okur et al., 2019). Notably, excessive addition of glycerol in films would lead to maceration of healthy skin around the wound area and other complications including infection, which is unfavorable (Boateng et al., 2013; Hafezi et al., 2019). This can be attributed to the leaching of glycerol from the film matrix and its humectant behavior (Pawar et al., 2013). Furthermore, it is noteworthy to highlight that there is as well a possibility of evaporation of plasticizers from the film matrix during storage (Suyatma et al., 2005). As reported in the literature, the evaporation rate of glycerol and PEG 400 molecules was found to be 0.3 mg/cm^2^.min and 0.88 µg/cm^2^.min, respectively (Benninghoven, 2012). This proves that the efficiency of glycerol as a plasticizer is lower, as its rate of evaporation and migration from the film matrix is relatively faster.

### Swelling studies

An ideal wound dressing should have the capacity to adequately handle wound exudate, maintain a moist environment, avoid inflammation and excess dehydration, be easy to use with little or no pain at the wound site, be cost-effective, and be cosmetically acceptable (Ma et al., 2017). Based on these, swelling behavior is an important parameter due to its significance in the practical application of wound dressings (Üstündağ Okur et al., 2018). Studies were conducted by soaking films in PBS (pH 7.4) to mimic the wound exudate on the wound surface (Hafezi et al., 2019). Visual observation indicated that formulated films swelled rapidly and expanded greatly in their size. The swelling capacity of the films was at maximum after 20 minutes of soaking in PBS. It was observed that the swelling of the films was rapid initially, followed by a slower decrease in the swelling rate until an equilibrium steady state was attained between 90 − 150 minutes. This tendency can be attributed to the abundant active hydroxyl groups that were unoccupied at the primary stage of the swelling process. The active sites were progressively saturated until they could no longer accommodate any fluid (Sanyang *et al.*, 2016). It is worth mentioning that the bio-composite films maintained their structural integrity throughout the swelling study.

ANOVA test was employed to assess the significant terms of the tested factor on the SI. Figure 2(e) displays the response surface plot of the impact of polymer concentration (X_1_) and plasticizer concentration (X_2_) on the SI of LNZ-loaded bio-composite films. The results display that only the plasticizer concentration (X_2_) had a significant impact on the SI (*p*-value < 0.0001). Rising the plasticizer concentration from 0% to 30% caused a significant rise in the SI. This is presumably due to plasticization which tends to form a large hydrodynamic complex with the swelling medium due to the hygroscopic nature of plasticizers, hence enhancing affinity with wounds (Jantrawut et al., 2017). Increasing the plasticizer concentration resulted in enhancing the interpenetration of the swelling medium to accommodate the interchain spacing, thus increasing the hydration rate of the films (Ediyilyam et al., 2021). Similar trend was reported in previous swelling studies, regarding the influence of the plasticizer concentration on the swelling behavior of the films (Ma et al., 2017; Bhagurkar *et al.*, 2019).

Figure 3(e) displays the impact of the plasticizer type on the SI of LNZ-loaded bio-composite films, where formulations containing glycerol resulted in higher SI compared to those containing PEG 400 (*p*-value = 0.0141). The effect of the plasticizer type on increasing the SI was more pronounced for glycerol than PEG 400. The hydrophilic nature of glycerol justifies this performance. The intrinsic characteristics of glycerol are mainly induced by liberating the abundant free amino groups to bind with fluids. Glycerol favored the dimensional stability by the development of a cross-linked network and increasing the affinity to the swelling medium by establishing the clustering effect. This result is in good agreement with the result of the moisture content, where glycerol resulted in increasing the moisture content, opening the polymer structure, increasing void volume and mobile regions, and hence increasing the sorption of more water in the film matrix. Furthermore, the polar surface of glycerol allows it to easily permeate into the polymer network and results in better wetting of the films (Ma et al., 2017). Similar investigations were reported for the enhanced SI of glycerol-plasticized films compared to PEG 400-plasticized films (Bajdik et al., 2009; Jung et al., 2016).

Previous studies reported that moderate to highly exudative wounds normally result in an average of 3 − 5 mL of exudate per 10 cm^2^ in 24 h. In our present study, 3 cm^2^ of the plasticized bio-composite films absorbed approximately 1659.70 ±1.38 – 2195.12 ±1.23% of the swelling medium. This reveals that the formulated dressings can absorb and retain a high volume of fluids, and thus are ideal for moderate to highly exudative wounds. Similar findings were reported by Boateng and colleagues for the glycerol-plasticized Polyox^TM^/carrageenan film loaded with streptomycin and diclofenac, where the film absorbed 2327% of the stimulating wound fluid (Boateng et al., 2013).

### Drug content determination

The drug content is a focal parameter due to its ability to study the potential of the drug delivery system to act as a carrier for the therapeutic agent. The percentages of drug content for the formulated bio-composite films are depicted in Table 3. The drug content was found to vary from 98.94 ± 0.001 to 99.93 ± 0.011%, with a low standard deviation. No significant difference in the drug contents was witnessed between the bio-composite films. This indicates that the film casting process was capable of giving reproducible results with uniform drug distribution in the polymeric matrix. Similar findings were reported by other authors (Bhagurkar et al., 2019; Üstündağ Okur et al., 2019).

### In vitro drug release

**Figure 4.**
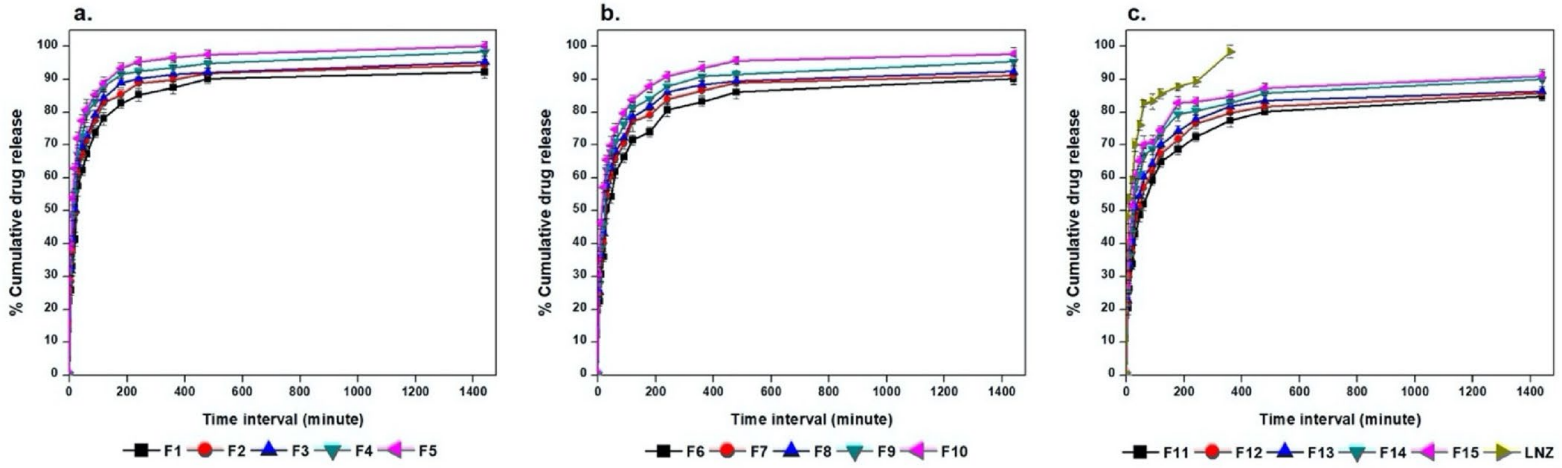
*In vitro* release studies of LNZ from a. chitosan/Tween 80, b. chitosan, and c. chitosan/gelatin bio-composite films in PBS (pH 7.4) at 32  ± 0.2 ^o^C for 24 h.

An ideal wound dressing should provide full drug release over a long period of time of 24 − 48 h (Hafezi et al., 2019). LNZ is a synthetic antibiotic with low aqueous solubility (Hedaya et al., 2017). The cumulative percentage of drug release from the films as well as the dissolution behavior of free LNZ were examined for 24 h and results are displayed in Figure 4. All LNZ-loaded bio-composite films reached almost complete dissolution after 24 h compared with that of the free drug after 6 h only. These results imply that the prepared films distinctively improved the extent and rate of dissolution of LNZ from the formulated LNZ-loaded bio-composite films. The release profile of LNZ from the films was characterized by an initial burst release within 30 minutes followed by a sustained release for about 24 h. The burst release was due to the rapid drug release from the superficial zones where the drug was loosely interacted or weakly bound with the film. This tendency is primarily advantageous to exert anti-bacterial action during the first few hours of the dressing application by drawing the mesenchymal cells into the films. Following this, sustained LNZ release was observed and can be attributed to the remaining drug fraction incorporated inside the film matrix which is merely released when the hydrated polymer starts to swell and hence drug diffusion takes place through the loose swollen matrix. This behavior avoids recolonization, necessity for frequent wound dressing changes, and promotes tissue remodeling in the subsequent stages of the healing process (Li et al., 2020). This can provide a potentially captivating opportunity for the formulated bio-composite films for regenerative medicine. The biphasic release can be attributed to the differences in the diffusion distances for drug molecules in the outer and inner matrix of polymeric films (Yaghoobian et al., 2020).

**Figure 5.**
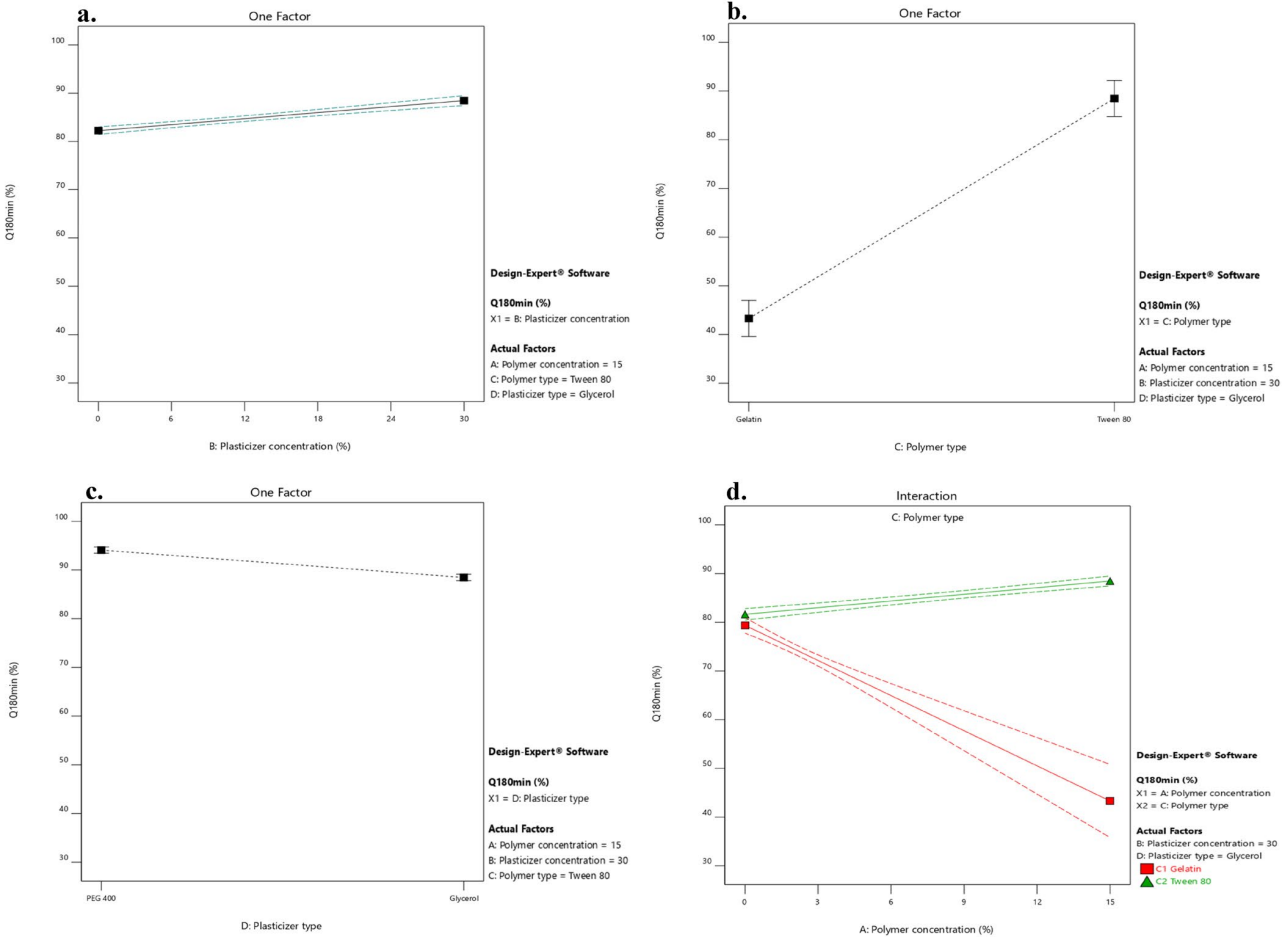
Impact of the independent variables and the interactions on the percentage of LNZ release after 180 minutes. a. X_2_: plasticizer concentration, b. X_3_: polymer type, c. X_4_: plasticizer type, and d. X_1_/X_3_: polymer concentration/polymer type.

ANOVA test was employed to assess the level of significance of the tested factors on the percentage of LNZ release from different bio-composite films after 30 and 180 minutes in addition to the interactions between these factors. Figure 5 displays the effect of the tested independent variables of plasticizer concentration (X_2_), polymer type (X_3_), and plasticizer type (X_4_) and the interaction between polymer concentration (X_1_)/polymer type (X_3_) on the percentage LNZ release from bio-composite films after 180 minutes (Y_7_) (similar responses were obtained after 30 minutes (Y_6_)).

A significant effect of the plasticizer concentration on the drug release was determined. Rising the plasticizer concentration from 0% to 30% caused a significant increase in the percentage of LNZ release after 30 and 180 minutes (*p*-value < 0.0001 for both variables). This effect can be attributed to the high aqueous miscibility of the plasticizers which results in opening the channels in the film matrix to enable solvent uptake, hence augmenting the drug release from the film matrix. Alike findings were reported by Pawar et al. and Azevedo et al. for Polyox^®^/carrageenan-loaded streptomycin and diclofenac films and chitosan-silver sulfadiazine loaded films, respectively (Azevedo et al., 2006; Pawar et al., 2013).

A significant effect of the polymer type on the drug release after 30 and 180 minutes was demonstrated, where formulations containing Tween 80 resulted in a higher percentage of LNZ released after 30 and 180 minutes compared to those containing gelatin (*p*-value < 0.0001 for both variables). This can be attributed to the solubilizing effect of the non-ionic surfactant Tween 80 that enhance the capability of the chitosan matrix to localize more fluids and facilitate drug release (Ediyilyam et al., 2021). Similar findings were reported by Bassi and Kaur and Akbari et al. for tamarind seed polysaccharide/Tween 60 film-loaded nystatin and solid dispersions of spironolactone with PEG and several types of Tween, respectively (Akbari et al., 2015; Bassi & Kaur, 2017). This result correlates well with the result of the swelling profile, where Tween 80, HLB of 15, resulted in increasing the swelling index, increasing wettability, increasing dissolution of the drug in the bio-composite films, and hence increasing the percentage of LNZ release after 30 and 180 minutes.

A significant effect of the plasticizer type on the drug release after 30 and 180 minutes was demonstrated, where formulations containing PEG 400 resulted in a higher percentage of LNZ released after 30 and 180 minutes compared to those containing glycerol (*p*-value < 0.0001 for both variables). The impact of the plasticizer type on enhancing the drug release was more pronounced for PEG 400 than glycerol. This can be ascribed to the hydrophilic nature of PEG 400, which upon dissolving results in the development of pores in the film structure and hence accelerates the drug release from the swollen film matrix. PEG 400 governs the rate of drug release from the film matrix *via* its sequential dissolution (Hafezi et al., 2019). Moreover, the solubilization effect of the efficient cosolvent PEG 400 improves the wettability, solubility, and dispersibility of the drug as well as enhancing the overall dissolution profile of the drug (Zaman & Hanif, 2018). This result correlates well with the result of the film thickness, where PEG 400 resulted in decreasing the film thickness, decreasing the distance of the drug diffusion across the polymer matrix, decreasing the lag time, and hence increasing the percentage of LNZ released after 30 and 180 minutes (Azevedo et al., 2006).

A significant influence of the effect of polymer concentration (X_1_)/polymer type (X_3_) on the drug release after 30 and 180 minutes was demonstrated (*p*-value < 0.0001 for both variables). Increasing the polymer concentration augmented with a superior extent the percentage of drug release from the film matrix containing Tween 80 compared to those containing gelatin. This is ascribed to the synergistic effect of polymer concentration and Tween 80 on augmenting the release of drug from the film matrix. Alternatively, the behavior of gelatin can be ascribed to the nominal expansion of the compact network of chitosan/gelatin films and the interactions between the drug and gelatin, which limit the drug diffusion across the polymeric network (Ma et al., 2017). Furthermore, the non-dissolved drug fraction dispersed in the film matrix acts as a reservoir, hence preserving the *Cs* value (concentration of saturated solution) constantly higher than the *C* value (concentration of solution). Accordingly, the *Cs*–*C* value was lower in the chitosan/gelatin films than in chitosan/Tween 80 films, which resulted in decreased drug release (Azevedo et al., 2006).

**Figure 6.**
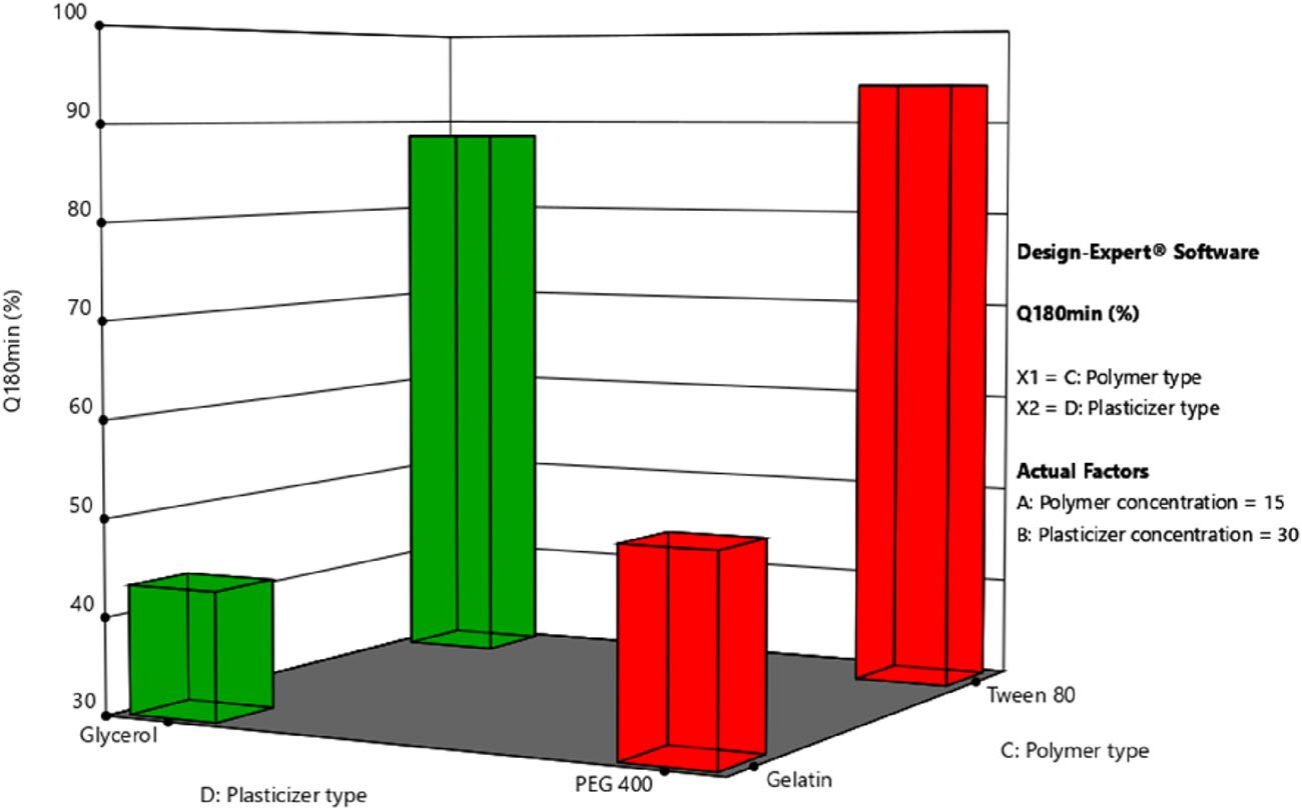
Response surface plot of the impact of the polymer type (X_3_)/plasticizer type (X_4_) on the percentage of LNZ release after 180 minutes.

Figures 2f and 6 displays the response surface plots of the impact of the polymer concentration (X_1_)/plasticizer concentration (X_2_) and the polymer type (X_3_)/plasticizer type (X_4_) on the percentage of LNZ release from bio-composite films after 180 minutes (Y_7_). It is evident that the maximum drug release was achieved at the highest levels of Tween 80 and PEG 400.

### Optimization

The main purpose of the experimental design is to figure out the suitable and optimum level of each independent variable (X_1_, X_2_, X_3_, and X_4_) that leads to the optimized formula. The optimum values of the responses were determined by the numerical optimization method using Design-Expert^®^ version 12.0.3.0 software. An ideal wound dressing should be flexible, maintain a moist environment, be capable of adequately handling wound exudate, possess adequate resistance, withstand mechanical stress, and provide a full drug release, accordingly the constraints were applied as follow: maximize thickness (Y_1_), moderate moisture content (Y_2_), minimize tensile strength (Y_3_), maximize elongation at break (Y_4_), maximize swelling index (Y_5_) and maximize percentage drug release after 30 and 180 minutes (Y_6_ and Y_7_, respectively). The optimized formulation was composed of 15% Tween 80 and 30% PEG 400, which has the highest desirability of 0.945. The selected bio-composite film would be efficient enough to maintain a moist environment for wound healing and adequately handle the wound exudate in accordance with its *in vitro* results. The optimized formula was further characterized for morphology, optical properties, thermal analysis, and microbial studies.

### Scanning electron microscopy (SEM)

**Figure 7.**
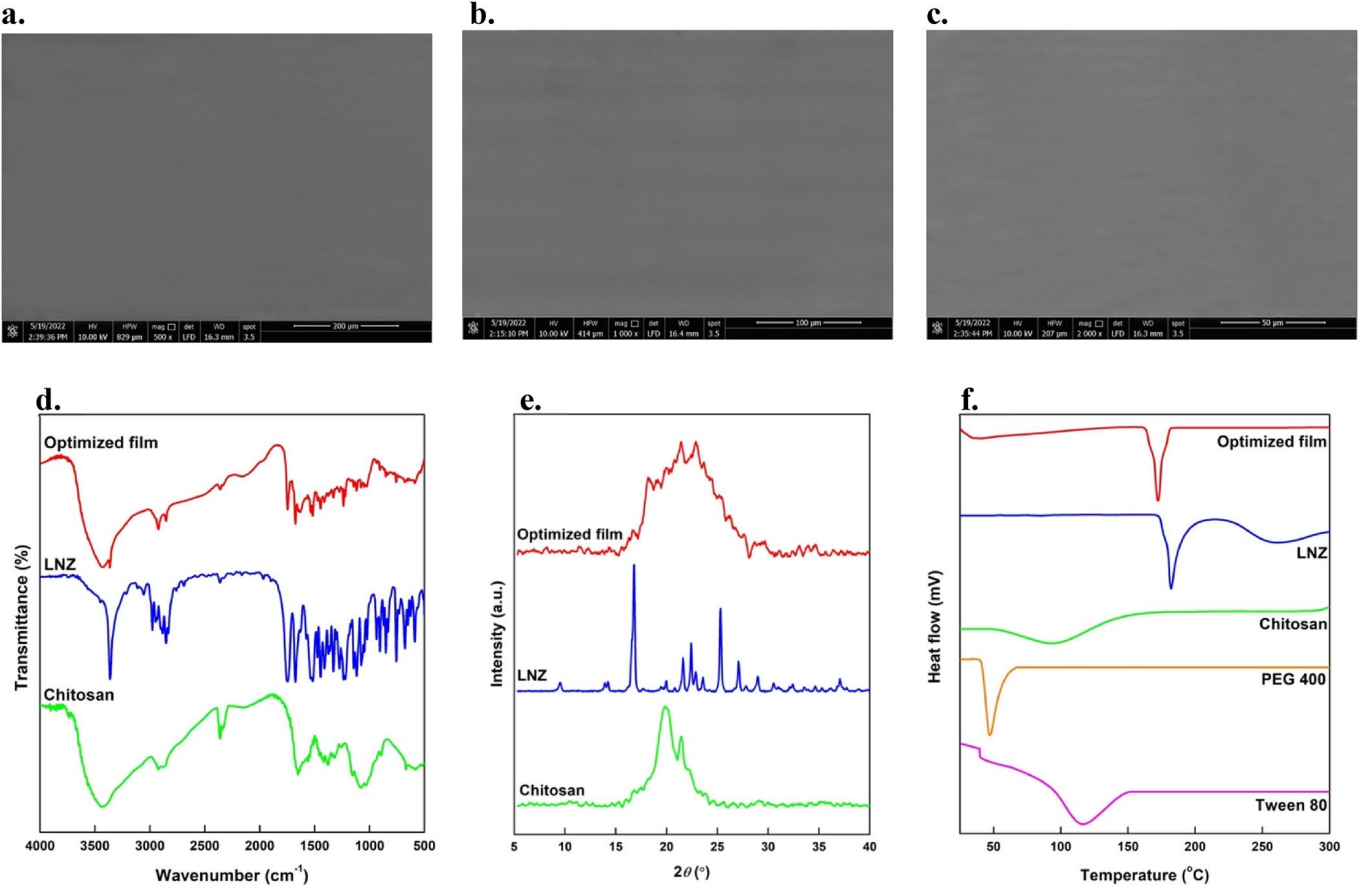
SEM images of the optimized bio-composite film at magnifications of a. 500, b. 1000, and c. 2000; d. FT-IR spectra and e. XRD thermograms of LNZ, chitosan, and the optimized bio-composite film; f. DSC thermograms of LNZ, chitosan, Tween 80, PEG 400, and the optimized bio-composite film.

SEM images of the optimized film at different magnifications are presented in Figure 7(a)–(c). The film showed a homogeneous, compact, and network structure with no visible pores or cracks on the film surface. This is a good indication of content uniformity, whereas chitosan, LNZ, Tween 80, and PEG 400 were homogeneously distributed within the bio-composite film. Moreover, this is mainly ascribed to the presence of the plasticizer PEG 400 that decreases the TS, while significantly enhancing the EB. Such flexibility of the film is ideal to decrease the probability of initiating further injury to the healing tissue (Hafezi et al., 2019). The observed surface features of the optimized bio-composite film verified the prior observations from the results of mechanical properties.

### Fourier-transform infrared spectroscopy (FT-IR)

The FT-IR spectra of LNZ, chitosan, and optimized bio-composite film are shown in Figure 7(d). The FT-IR spectrum of chitosan showed characteristic bands of –OH broad band that overlaps –NH stretching at 3500–3400 cm^−1^, aliphatic –CH stretching at 2923 and 2878 cm^−1^, carbonyl (C = O) stretching of amide I at 1651 cm^−1^ and –OH bending of alcohol groups (–CH_2_–OH) at 1377 cm^−1^. The bands at 1154, 1072, 1033, and 895 cm^−1^ result from the saccharide structure of chitosan in the fingerprint region. While the FT-IR spectrum of LNZ revealed characteristic bands of –NH group at 3361 cm^−1^, aromatic –CH at 3057 cm^−1^, aliphatic –CH at 2978 and 2853 cm^−1^, C = O of an inner ester of five-membered ring and amide at 1747 and 1675 cm^−1^, respectively. Other researchers reported alike FT-IR spectra for chitosan and LNZ pure compounds (Bakr *et al.*, 2021; Shah et al., 2022).

The FT-IR spectrum of the optimized bio-composite film of LNZ and chitosan demonstrated that all characteristic bands were retained in the film, and hence the polymer and drug were compatible with each other. The C=O stretching of amide I of the optimized bio-composite film shifted to a lower wavelength (1629 cm^−1^). Typically, this reduction in the vibrational wavelength is indicative of inter-molecular hydrogen bonds among the chitosan structure. Furthermore, once the interaction between the –NH of the drug and the –OH of chitosan occurred after solvent casting, the band at 1377 cm^−1^ shifted to 1328 cm^−1^. No new vibration bands were observed indicating the stability of the optimized bio-composite film (Üstündağ Okur et al., 2019). Therefore, FT-IR analysis revealed that the successful incorporation of LNZ and chitosan into the bio-composite film resulted in different molecular interactions in the film matrix *via* physical interactions of hydrogen bonding rather than chemical reactions (Ma et al., 2017).

### Differential scanning calorimetry (DSC)

The DSC thermograms of LNZ, chitosan, Tween 80, PEG 400, and optimized bio-composite film were shown in Figure 7(f). LNZ showed a sharp endothermic peak near 182 °C, which corresponds to its melting point and crystalline nature (Shah et al., 2022). The broad endothermic peaks near 95 °C and 116 °C were observed in the DSC curves of chitosan and Tween 80, respectively, which correspond to their amorphous nature and flash points. Whereas PEG 400 showed an endothermic peak near 47 °C, as seen in Figure 7(f). The DSC thermograms of LNZ, chitosan, Tween 80, and PEG 400 were in reasonable agreement with the reported data in the literature (Ashfaq et al., 2022; Shah et al., 2022). The DSC curve of the optimized bio-composite film showed a sharp endothermic peak near 173 °C, which confirms the presence of LNZ without any change in its physical form, thus reaffirming that the drug and polymers are compatible with each other (Shah et al., 2022). The slight decrease in the melting point of LNZ in the optimized bio-composite film may be due to the presence of the plasticizer PEG 400, which can reduce the transition temperature of materials. Furthermore, it can be attributed to the transformation of the crystalline LNZ into an amorphous form. Therefore, it can be concluded from the results that LNZ displayed thermal stability both in the powder form and in the bio-composite film formulation (Zaman & Hanif, 2018).

### X-ray diffraction (XRD)

Diffractograms of chitosan, pure LNZ, and optimized film are shown in Figure 7(e). The diffraction peaks notably at 19.8° and 21.4° are the characteristic fingerprints for pure chitosan and ascribed to a more amorphous structure (Liu et al., 2012). XRD studies showed that pure LNZ was a crystalline drug with various sharp distinct peaks at 2*θ* diffraction angles of 9.5°, 14.2°, 16.8°, 17.7°, 21.6°, 22.4°, 25.3°, 27.0° and 29.0°. However, in the optimized bio-composite film formulation, the crystallinity of LNZ was masked. The intensity of the diffraction peaks in the optimized film becomes broad and comparatively less intense demonstrating that the presence of chitosan resulted in reducing the crystallinity of LNZ, hence transitioning the crystalline drug into amorphous (Zaman & Hanif, 2018). This tendency is attributed to the hydrogen bonding interaction between LNZ and chitosan. The results correlate well with the data mentioned in the FT-IR studies. Of interest, no considerable change in the d-spacing values was detected, thus suggesting no change in the crystal form of LNZ, however, its crystal habit may be changed. The results are in good agreement with the literature studies (Zaman & Hanif, 2018).

### In vitro antibacterial activity by disc diffusion method

**Figure 8.**
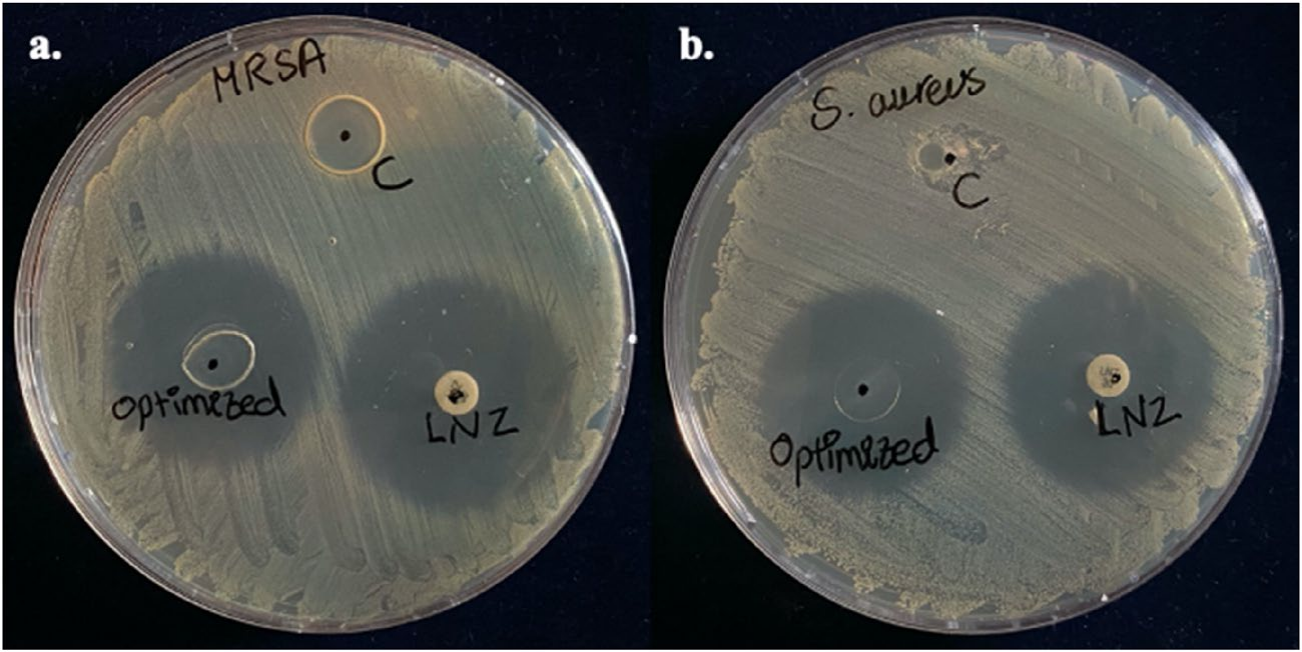
Antibacterial activity of pure chitosan (C), LNZ disc, and the optimized bio-composite film against a. MRSA and b. *Staphylococcus aureus* (ATCC^®^ 25922).

The antibacterial activity of pure chitosan, LNZ disc, and the optimized film was detected as ZOI surrounding the discs against MRSA and *Staphylococcus* aureus (ATCC^®^ 25922) (Üstündağ Okur et al., 2019). The diameters of ZOI for LNZ disc and optimized bio-composite film against *Staphylococcus* aureus (ATCC^®^ 25922) were alike (30 mm), while against MRSA were comparable to 32 mm and 30 mm, respectively, whereas chitosan control film did not show any ZOI, as shown in Figure 8 and Table S1. It is widely known that pure chitosan possesses distinctive antibacterial activity because of its cationic property. Of interest, this seemingly different result for pure chitosan is mostly because of the limits of detection of its antibacterial activity while using the disc diffusion method (Reddy *et al.*, 2016). Similar results were reported by other researchers (Reddy *et al.*, 2016; Ma *et al.*, 2017). The results correlate well with the data mentioned in the *in vitro* drug release studies, indicating that the optimized film-loaded LNZ can diffuse out easily and completely within 24 h. Therefore, the optimized film possesses antibacterial activity and has the potential for use as a wound dressing to reduce the bacterial load and prevent wound colonization and thus improve wound healing.

### Sterility test for bio-composite films

Both control and optimized bio-composite films showed no growth in the broth medium, which indicates the sterility of the films. Therefore, the optimized film formulation was conforming with the requirements of the official monographs of the British Pharmacopeia for the lack of both *Staphylococcus aureus* and *Pseudomonas aeruginosa* (Ahmed et al., 2018).

## Conclusion

A novel biphasic release bio-composite film of LNZ which possesses properties that meet desirable characteristics of an ideal dressing was successfully prepared, characterized, and optimized using response surface methodology. A mathematical model was developed in order to link the significant formulation variables along with the measured responses. The optimized LNZ-loaded bio-composite film, composed of 15% Tween 80 and 30% PEG 400, using chitosan as a matrix former possessed the most desirable properties. It was able to release 100 ± 0.07% of LNZ with satisfactory release characteristics. The optimized LNZ-loaded bio-composite film also exhibited distinctive antibacterial activity against MRSA and *Staphylococcus aureus*, which are among the most common bacteria responsible for wound infections. Therefore, the developed LNZ-loaded bio-composite film can be proposed as a promising antimicrobial dressing for potential use in the treatment of skin injuries to help to achieve rapid wound healing.

## Supplementary Material

Supplemental Material

## Data Availability

Not applicable.
