# Supplementary material for: Novel linezolid loaded bio-composite films as dressings for effective wound healing: experimental design, development, optimization, and antimicrobial activity
Source: Drug Deliv. 2022 Oct 2;29(1):3168–85. doi: 10.1080/10717544.2022.2127974 (PMC9543119; doi:10.1080/10717544.2022.2127974)
Supplement: Supplemental Material [file IDRD_A_2127974_SM1284.docx]

***List of Supplementary Tables***

| **Supplementary table number** | **Caption** |
| --- | --- |
| Table S1 | Antibacterial activity of pure chitosan, LNZ disc, and the optimized bio-composite film against MRSA and *Staphylococcus aureus* (ATCC^®^ 25922) expressed in terms of diameter of zone of inhibition. |
| Table S2 | Evaluation parameters and formulations of I-optimal design matrix and their experimental values of responses for the LNZ-loaded bio-composite films. |

**Table S1.** Antibacterial activity of pure chitosan, LNZ disc, and the optimized bio-composite film against MRSA and *Staphylococcus aureus* (ATCC^®^ 25922) expressed in terms of diameter of zone of inhibition.

| Microorganism tested | Diameter of microbial zone of inhibition (mm) | | |
| --- | --- | --- | --- |
|  | Pure chitosan | LNZ disc (30µg) | Optimized film (30µg) |
| MRSA | 0 | 32 (S) | 30 (S) |
| *Staphylococcus aureus* (ATCC^®^ 25922) | 0 | 30 (S) | 30 (S) |

S: susceptible.

**Table S2.** Evaluation parameters and formulations of I-optimal design matrix and their experimental values of responses for the LNZ-loaded bio-composite films.

| Run | X_1_:  Polymer concentration (%) | X_2_: Plasticizer concentration (%) | X_3_:  Polymer type | X_4_:  Platicizer type | Y_1_ (mm) | Y_2_  (%) | Y_3_  (MPa) | Y_4_  (%) | Y_5_  (%)^a^ | Y_6_  (%) | Y_7_ (%) | Weight variation (g) | Surface pH | DC  (%)^b^ |
| --- | --- | --- | --- | --- | --- | --- | --- | --- | --- | --- | --- | --- | --- | --- |
| 1 | 15 | 15 | Tween 80 | Glycerol | 0.202 ±0.02 | 18.89 ±1.57 | 18.93 ±1.20 | 61.11 ±3.91 | 2004.44 ±2.55 | 60.92 ±0.06 | 85.28 ±0.07 | 0.045 ±0.001 | 5.40 ±0.01 | 99.52 ±0.006 |
| 2 | 0 | 30 | Tween 80 | PEG 400 | 0.159 ±0.02 | 13.75 ±1.06 | 6.01 ±0.81 | 78.46 ±1.50 | 2010.63 ±4.13 | 65.31 ±0.04 | 87.77 ±0.09 | 0.040 ±0.001 | 5.45 ±0.04 | 99.27 ±0.001 |
| 3 | 2 | 30 | Gelatin | PEG 400 | 0.170 ±0.01 | 15.66 ±0.68 | 6.13 ±1.13 | 70.17 ±1.79 | 2022.89 ±1.42 | 60.35 ±0.04 | 82.85 ±0.02 | 0.042 ±0.000 | 5.50 ±0.03 | 99.70 ±0.001 |
| 4 | 15 | 30 | Tween 80 | PEG 400 | 0.206 ±0.02 | 19.59 ±2.04 | 5.19 ±2.89 | 88.18 ±3.02 | 2047.42 ±1.99 | 71.92 ±0.08 | 93.42 ±0.06 | 0.048 ±0.001 | 5.48 ±0.02 | 99.86 ±0.001 |
| 5 | 15 | 15 | Tween 80 | PEG 400 | 0.146 ±0.01 | 14.29 ±0.37 | 16.42 ±2.37 | 79.74 ±2.01 | 1845.45 ±3.31 | 67.14 ±0.06 | 91.54 ±0.05 | 0.038 ±0.001 | 5.42 ±0.01 | 99.77 ±0.001 |
| 6 | 2 | 0 | Gelatin | Glycerol | 0.099 ±0.02 | 10.91 ±0.51 | 61.71 ±5.09 | 8.24 ±1.11 | 1537.27 ±2.87 | 42.81 ±0.01 | 68.61 ±0.03 | 0.028 ±0.001 | 5.40 ±0.04 | 99.17 ±0.001 |
| 7 | 15 | 0 | Tween 80 | Glycerol | 0.125 ±0.02 | 13.73 ±0.42 | 53.00 ±3.81 | 21.38 ±1.85 | 1615.64 ±5.23 | 57.34 ±0.06 | 82.55 ±0.06 | 0.033 ±0.002 | 5.40 ±0.01 | 99.56 ±0.001 |
| 8 | 2 | 15 | Gelatin | PEG 400 | 0.114 ±0.02 | 11.77 ±0.42 | 18.03 ±2.64 | 60.22 ±3.97 | 1778.68 ±3.36 | 56.72 ±0.02 | 79.26 ±0.05 | 0.034 ±0.001 | 5.48 ±0.02 | 99.64 ±0.001 |
| 9 | 0 | 0 | Gelatin | Glycerol | 0.088 ±0.01 | 10.21 ±1.15 | 56.92 ±5.29 | 12.67 ±1.14 | 1309.18 ±4.60 | 50.45 ±0.01 | 73.97 ±0.04 | 0.025 ±0.001 | 5.40 ±0.01 | 98.94 ±0.001 |
| 10 | 0 | 30 | Tween 80 | Glycerol | 0.184 ±0.01 | 17.35 ±0.87 | 10.52 ±3.11 | 45.73 ±4.14 | 2077.55 ±2.12 | 57.63 ±0.03 | 81.62 ±0.02 | 0.049 ±0.001 | 5.50 ±0.01 | 99.40 ±0.001 |
| 11 | 0 | 15 | Tween 80 | Glycerol | 0.122 ±0.02 | 12.86 ±1.21 | 20.12 ±1.75 | 57.32 ±5.57 | 1903.57 ±2.10 | 54.41 ±0.04 | 79.02 ±0.01 | 0.035 ±0.002 | 5.47 ±0.03 | 99.93 ±0.011 |
| 12 | 2 | 0 | Gelatin | Glycerol | 0.096 ±0.02 | 10.55 ±0.51 | 61.32 ±5.09 | 9.40 ±1.11 | 1539.29 ±2.87 | 42.81 ±0.01 | 68.53 ±0.03 | 0.029 ±0.001 | 5.44 ±0.04 | 99.17 ±0.001 |
| 13 | 15 | 0 | Tween 80 | Glycerol | 0.128 ±0.02 | 13.43 ±0.42 | 51.32 ±3.81 | 20.12 ±1.85 | 1611.94 ±5.23 | 57.43 ±0.06 | 81.64 ±0.06 | 0.034 ±0.001 | 5.41 ±0.01 | 99.55 ±0.001 |
| 14 | 2 | 15 | Gelatin | Glycerol | 0.125 ±0.01 | 13.16 ±0.37 | 22.17 ±4.49 | 49.67 ±3.51 | 1931.58 ±5.06 | 46.90 ±0.01 | 71.58 ±0.02 | 0.038 ±0.001 | 5.46 ±0.01 | 99.92 ±0.001 |
| 15 | 0 | 15 | Tween 80 | Glycerol | 0.123 ±0.02 | 12.00 ±1.21 | 21.18 ±1.75 | 59.87 ±3.57 | 1905.06 ±2.10 | 54.34 ±0.04 | 79.00 ±0.01 | 0.037 ±0.002 | 5.44 ±0.03 | 99.92 ±0.011 |
| 16 | 0 | 15 | Gelatin | PEG 400 | 0.095 ±0.02 | 10.45 ±0.84 | 17.26 ±4.51 | 69.75 ±1.08 | 1659.70 ±1.38 | 62.43 ±0.03 | 84.01 ±0.03 | 0.034 ±0.002 | 5.42 ±0.04 | 99.24 ±0.001 |
| 17 | 2 | 30 | Gelatin | Glycerol | 0.211 ±0.03 | 20.76 ±1.07 | 14.75 ±1.49 | 41.94 ±2.44 | 2140.57 ±0.95 | 50.74 ±0.01 | 74.25 ±0.01 | 0.053 ±0.001 | 5.49 ±0.01 | 99.14 ±0.001 |
| 18 | 2 | 30 | Gelatin | PEG 400 | 0.166 ±0.01 | 16.14 ±0.68 | 6.41 ±1.13 | 69.56 ±1.79 | 2023.90 ±1.42 | 60.29 ±0.04 | 82.73 ±0.02 | 0.043 ±0.000 | 5.47 ±0.03 | 99.71 ±0.001 |
| 19 | 15 | 30 | Tween 80 | Glycerol | 0.239 ±0.02 | 24.40 ±0.46 | 9.11 ±3.18 | 57.67 ±2.31 | 2195.12 ±1.23 | 63.84 ±0.08 | 88.83 ±0.10 | 0.062 ±0.001 | 5.44 ±0.01 | 99.22 ±0.014 |

**Note:** Results are mean values of three replicates and the overall standard deviation values were < ±5.5.

**^a^** Swelling index values after 20 minutes. **^b^** DC: Drug content.
